# Effects of Troponin T Cardiomyopathy Mutations on the Calcium Sensitivity of the Regulated Thin Filament and the Actomyosin Cross-Bridge Kinetics of Human β-Cardiac Myosin

**DOI:** 10.1371/journal.pone.0083403

**Published:** 2013-12-18

**Authors:** Ruth F. Sommese, Suman Nag, Shirley Sutton, Susan M. Miller, James A. Spudich, Kathleen M. Ruppel

**Affiliations:** 1 Department of Biochemistry, Stanford University School of Medicine, Stanford, California, United States of America; 2 Department of Pharmaceutical Chemistry, University of California San Francisco, San Francisco, California, United States of America; 3 Department of Pediatrics (Cardiology), Stanford University School of Medicine, Stanford, California, United States of America; Tokyo Medical and Dental University, Japan

## Abstract

Hypertrophic cardiomyopathy (HCM) and dilated cardiomyopathy (DCM) lead to significant cardiovascular morbidity and mortality worldwide. Mutations in the genes encoding the sarcomere, the force-generating unit in the cardiomyocyte, cause familial forms of both HCM and DCM. This study examines two HCM-causing (I79N, E163K) and two DCM-causing (R141W, R173W) mutations in the troponin T subunit of the troponin complex using human β-cardiac myosin. Unlike earlier reports using various myosin constructs, we found that none of these mutations affect the maximal sliding velocities or maximal Ca^2+^-activated ADP release rates involving the thin filament human β-cardiac myosin complex. Changes in Ca^2+^ sensitivity using the human myosin isoform do, however, mimic changes seen previously with non-human myosin isoforms. Transient kinetic measurements show that these mutations alter the kinetics of Ca^2+^ induced conformational changes in the regulatory thin filament proteins. These changes in calcium sensitivity are independent of active, cycling human β-cardiac myosin.

## Introduction

Hypertrophic cardiomyopathy (HCM) and dilated cardiomyopathy (DCM) are heritable cardiac disorders that are significant causes of heart failure, arrhythmias, and sudden cardiac death worldwide. HCM affects approximately 1 in 500 individuals, and is characterized by thickening of the left ventricular heart wall, reduced left ventricular chamber volume, fibrosis, and cardiomyocyte disarray [1], [2]. Clinically, patients with HCM typically have preserved or even enhanced global contractile or systolic function, but impaired relaxation or diastolic function. Specific disease-causing mutations in genes encoding sarcomeric proteins have been identified in at least 50% of HCM cases [3]. On the other hand, DCM is a leading indication for cardiac transplantation in both adult and pediatric populations. The pathophysiology of DCM involves thinning of one or both heart walls and enlargement of the left ventricular chamber, and is characterized clinically by systolic dysfunction [4]. DCM frequently occurs secondary to other insults, but ≥ 20% has been estimated to have a genetic cause [5].

Since mutations in the myosin heavy chain 7 gene (*MHY7*) have been linked to a significant number of familial cardiomyopathies, much work has been focused on the contractile properties of β-cardiac myosin [6]. For the heart to function properly, however, the regulation of contraction and relaxation by means of Ca^2+^ is critical [7], [8]. Together, actin, tropomyosin, and the troponin complex, also known as the thin filament, render the actin-myosin interaction sensitive to Ca^2+^. Mutations in the thin filament proteins have been linked to ∼5–10% of both HCM and DCM cases [9]–[11].

The troponin complex is the Ca^2+^ sensor in the muscle and is composed of three subunits: troponin C (TnC) that binds Ca^2+^, troponin I (TnI) that is the inhibitory subunit, and troponin T (TnT) that binds tropomyosin and communicates the Ca^2+^ signal from TnC to tropomyosin [12]. In the absence of Ca^2+^, tropomyosin is locked in a position along the actin filament that blocks the binding of myosin. Upon Ca^2+^ binding to TnC, the inhibitory region of TnI dissociates from tropomyosin allowing the tropomyosin to move azimuthally on the actin filament revealing the myosin binding sites on actin. Binding of the myosin to the thin filament is believed to further push the tropomyosin and open up the filament for myosin binding [13]. Mutations in these proteins, particularly the troponin complex, have been studied using various methods, such as animal models, skinned fibers into which mutant proteins have been exchanged, and reconstituted in vitro molecular studies such as ATPase, fluorescence, and motility assays (e.g. [14], [15]).

In vitro molecular studies are critical for laying the foundation for the effects of such mutations on the fundamental contractile apparatus. Extensive studies by many investigators have yielded important advances in understanding the effects of HCM- and DCM-causing mutations on a reconstituted six-component system consisting of actin, myosin, tropomyosin and the three troponin subunits (for reviews, see [12], [16]–[19]). Prior to this current study, however, all biochemical thin filament studies had been performed using non-human cardiac myosin (e.g. [20]–[22]) or with skeletal myosin (e.g. [15], [23]–[25]), as there was no expression system for active, human β-cardiac myosin. In the motor domain alone, those cardiac isoforms differ from human β-cardiac myosin by >30 amino acids, while the skeletal myosins differ by >150 residues. While it is unlikely that the major paradigms emanating from earlier work would be changed by using human β-cardiac myosin, it is important to establish that this is indeed true. Furthermore, details of specific kinetic steps in the chemomechanical cycle and operation of the six-component system are very likely to be changed somewhat by replacing non-human myosins with human β-cardiac myosin, and HCM- and DCM-causing mutations are expected to induce very small changes in such parameters, resulting in the diseased state. Thus, these details need to be fully delineated to have a rigorous and complete understanding of how the system operates. It is therefore important to begin to examine a six-component system that uses the human β-cardiac myosin as the motor.

Recently, using the C2C12 myoblast expression system developed by Winkelmann and colleagues [26] and adapted to the cardiomyopathy problem by Leinwand and colleagues, it has become possible to express and purify active human β-cardiac myosin [27], [28]. This study is the first to examine the role of human β-cardiac myosin on both the inherent Ca^2+^ sensitivity of the thin filament and the effect of these mutations on maximal Ca^2+^-activated ADP release kinetics.

**Figure 1:**
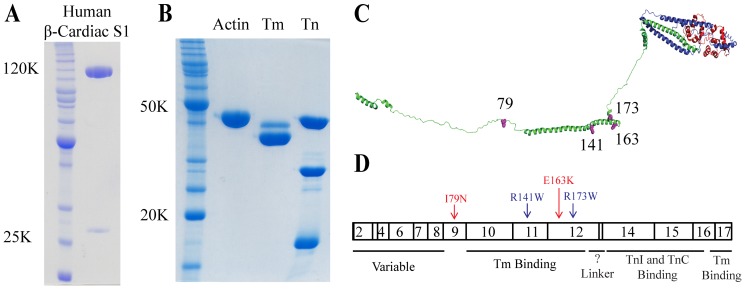
Expression of recombinant human β-cardiac S1 and thin filament proteins. (A) *Lane 1*, an Invitrogen Benchmark Ladder; *Lane 2*, WT human β-cardiac S1 (∼120 kDa) with a C-terminal GFP tag and the FLAG-tagged human ventricular essential light chain (ELC) (∼24 kDa). (B) *Lane 1*, an Invitrogen Benchmark Ladder; *Lane 2*, chicken skeletal actin (∼42 kDa); *Lane 3*, bovine tropomyosin (Tm; α isoform lower and β isoforms upper, ∼33 kDa); and *Lane 4*, human cardiac troponin complex (Tn; TnT ∼35 kDa, TnI ∼24 kDa, TnC ∼18 kDa). (C) A model of the troponin complex which is built from partial crystal structures of the human cardiac troponin complex of TnT (green), TnI (blue), and TnC (red) (Adapted from [72], PDB 1J1E). Mutations are shown in magenta. (D) Human cardiac TnT exons and functional domains [16]. HCM-causing mutations are in red and DCM-causing mutations in blue.

Here, we address the mechanism of Ca^2+^ sensitivity of two HCM- and two DCM-causing TnT mutations, using recombinant single-headed human β-cardiac myosin S1 (Subfragment 1) (Fig. 1). The two HCM-causing TnT mutations selected were E163K and I79N. E163K has been shown to increase the Ca^2+^ sensitivity in porcine cardiac skinned fibers, though little is known in a biochemically reconstituted system [20]. Thus, this study is one of the first to characterize this mutation with a six-component biochemically reconstituted system. I79N, on the other hand, is one of the most investigated TnT mutations, having been identified and described in the mid-1990s [29], [30]. An early study on I79N reported no change in the Ca^2+^ sensitivity [24], but subsequent studies in both fibers and in reconstituted systems reported increases in Ca^2+^ sensitivity [14], [20], [31]–[33]. Using a soluble subfragment of rabbit skeletal myosin containing both enzymatic heads or HMM, I79N was also shown to increase the maximal sliding velocity under low load [24]. Of the two DCM mutations, R173W has only recently been identified and has not been examined biochemically or in fibers [34]. R141W, on the other hand, has been studied in both porcine and rabbit cardiac fibers and with rabbit skeletal myosin, and was shown to have either the same Ca^2+^ sensitivity as wild type (WT) or a slight desensitization [15], [21], [22], [35]. It was also shown, using rabbit skeletal HMM, to have decreased maximal sliding velocity under low load [15].

Overall, our results agree with the paradigm established by earlier studies that HCM-causing mutations are sensitizing while DCM-causing mutations are desensitizing. Our work also shows that the presence of cycling motor has no effect on the Ca^2+^ sensitivity of the thin filaments under the conditions of our experiments. Indeed, kinetic analysis of these mutations shows that the rates of the conformational changes in the regulatory proteins are altered in a way that is consistent with the changes in Ca^2+^ sensitivity of all the mutants. When examining the effects of these mutations on myosin ADP release rates, however, our results differ from those obtained earlier. As mentioned above, previous studies using rabbit skeletal HMM have reported changes in maximally activated in vitro motility velocities under low load for a number of mutant thin filaments (suggesting changes in myosin ADP release kinetics), including a few we chose to examine in our study. In our study using human β-cardiac S1, however, we find no changes in maximally activated in vitro motility velocities under low load for any of the TnT mutations examined. This is an important result because velocity is a reflection of the step size and strongly bound state time of the myosin, which are key parameters affecting force production. Overall, our results show that one can now examine subtle effects of HCM- and DCM-causing mutations on the behavior of the six-component system using human β-cardiac myosin.

## Experimental Procedures

### Cloning of human cardiac troponin mutants and human β-cardiac myosin

The cDNAs for human adult cardiac TnI, TnC, and TnT in carbenicillin selective pET-3d plasmids were obtained as a gift from Dr. James Potter, University of Miami. I79N, R141W, E163K, and R173W mutations were introduced by QuikChange site-directed mutagenesis (Stratagene) into TnT and confirmed by sequencing. For the fluorescence studies, a triple mutation of TnC was made containing the mutations C35S, T53C, and C84S [36] using the same strategy. Human myosin heavy chain 7 (*MHY7*) cDNA and human ventricular essential light chain (*MYL3*; ELC) were purchased from Open Biosystems (Thermo). A truncated version of *MHY7* (residues 1-808), corresponding to a short human S1 motor domain with the human ELC, was constructed and produced as previously described [37]. Constructs were made with (for ATPase and motility, Fig. 1A) and without (for stopped flow, where fluorescence from the eGFP interferes) a C-terminal eGFP. For the stopped flow motor construct, the eGFP was replaced with a short non-fluorescent eight amino acid peptide [38], which we have developed for a separate project.

### Troponin expression and purification

Human adult cardiac troponin subunit (*TNNT2*, *TNNI3*, *TNNC1*) expression, purification, and complex formation protocols were based on previously published methods [39]–[41]. Bacterial pellets were resuspended in Lysis Buffer (6 M Urea, 50 mM Tris, pH 7.5, 2 mM EDTA, and 1 mM DTT) supplemented with PMSF and protease inhibitors (Roche). The bacteria were lysed by sonication and clarified by spinning at 25,000×g for 45 minutes at 4°C. Lysate was then run over ion exchange columns at 4°C using a 0–0.6 M KCl gradient of the Lysis Buffer. A 5 mL HiTrapQ column was used for TnC, and a 5 mL HiTrapS column was used for TnT and TnI (GE Healthcare).

For TnT, the peak fractions were dialyzed overnight in Lysis Buffer without KCl, and then run over a 5 mL HiTrapQ column (GE Healthcare). The cleanest fractions were dialyzed first into 20 mM Imidazole, pH 7.5, 3 M Urea, 1 M KCl, 1 mM MgCl_2_, and 1 mM DTT and then into Storage Buffer (20 mM Imidazole, pH 7.5, 1 M KCl, 1 mM MgCl_2_, and 1 mM DTT). TnT was flash frozen at >2 mg/ml and stored at −80°C for future use, with typical yields ∼20 mgs per L of bacteria culture. For TnC, peak fractions were dialyzed into PS Buffer (50 mM Tris, pH 7.5, 1 mM CaCl_2_, 1 mM MgCl_2_, 50 mM NaCl, and 1 mM DTT) at 4°C overnight. After dialysis, ammonium sulfate was added to a concentration of 0.5 M at 23°C and the dialysate was loaded onto a 5 mL phenylsepharose column equilibrated in PS Buffer at 23°C. Protein was eluted with 50 mM Tris, pH 7.5, 1 mM EDTA, and 1 mM DTT. Peak fractions were dialyzed into Storage Buffer, flash frozen, and stored at −80°C, with typical yields of ∼40–50 mgs per L of bacteria culture. For TnI, collected fractions were dialyzed into Affinity Buffer (50 mM Tris, pH 7.5, 2 mM CaCl_2_, 1 M NaCl, and 1 mM DTT) overnight at 4°C. After a brief spin to remove precipitated material, the dialysate was loaded onto a prepared 5 mL TnC affinity column and eluted using a linear gradient from Affinity Buffer to Elution Buffer (50 mM Tris, pH 7.5, 6 M Urea, 1 mM EDTA, and 1 mM DTT). The purest fractions were dialyzed into Storage Buffer, flash frozen, and stored at −80°C. Typical yields were ∼10 mgs per L of bacteria culture.

Complexes were formed according to Szczesna et al. with slight modifications [20]. Components were mixed at molar ratios of 1.3 TnI:1.3 TnT:1 TnC for one hour on ice. Complexes were then dialyzed at 4°C in six sequential steps into Complex Buffer (20 mM Imidazole, pH 7.5, 2 mM MgCl_2_, and 1 mM DTT) containing 0.7 M, 0.5 M, 0.3 M, 0.1 M, and 0.01 M KCl twice, for 6–12 hours each. Precipitated TnI and TnT was removed from the final complex solution by centrifugation for 10 minutes at 2,000×g. Troponin complexes were flash frozen at ∼1–2 mg/mL and stored at −80°C (Fig. 1B).

### Actin, myosin and tropomyosin purification

Actin was prepared from fresh chicken breast skeletal muscle as previously described [37], [42]. A detailed procedure for expressing and purifying human β-cardiac S1 is described elsewhere [27], [37]. Tropomyosin was purified from bovine cardiac tissue according to the protocol of Smillie, with a few modifications [43]. First, tropomyosin was extracted twice (overnight and then for two hours) from bovine cardiac acetone powder at 4°C with stirring in 1 M KCl, 25 mM Tris, pH 8.0, 0.1 mM CaCl_2_, and 0.1 mM DTT. Second, the tropomyosin pellet after the 65% ammonium sulfate precipitation was dissolved into HA Buffer (10 mM sodium phosphate buffer, 1 M KCl, 0.25 mM DTT). Tropomyosin was then dialyzed with 2–3 buffer changes against HA Buffer before it was loaded onto a 10 ml hydroxyapatite column and eluted with a linear gradient of 10 to 300 mM sodium phosphate. The peak fractions were dialyzed into 20 mM Imidazole, pH 7.5, 300 mM KCl, and 1 mM DTT before flash freezing and storing at −80°C (Fig. 1B). Typical yields were ∼5–10 mgs per gram of acetone powder.

### Coupled actin-activated ATPase assay

To determine the steady state actin-activated ATPase rate for the S1-thin filament complexes under different Ca^2+^ concentrations we used the NADH-coupled assay [44]. The assay was performed with freshly prepared S1 and actin at 30°C. The concentrations used were 3.5 µM actin, 1 µM tropomyosin, 2 µM troponin complex, and 0.3 to 0.5 µM S1. The final buffer conditions were 20 mM Imidazole (pH 7.5), 10 mM KCl, 2 mM MgCl_2_, 1 mM DTT, 2 mM ATP, the Ca^2+^ buffers (2 mM EGTA, 4 mM NTA, and varying concentrations of CaCl_2_), and the NADH-coupling system [44]. The Ca^2+^ buffers with EGTA and NTA were calculated using the pCa calculator developed by Dweck et al. [45]. These pCa values do not take into account the buffering capacity of the free troponin complex in solution. All buffers were carefully adjusted to be pH 7.5 at 30°C in the final reactions, and data were fit to the Hill equation (Eqn. 1) using SigmaPlot to determine the pCa50, the maximum (y_max_) and minimum (y_min_) activity, and the Hill coefficient (*n_H_*). 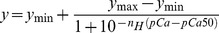
(Eqn.1)


For each S1 preparation, average maximal WT activity was considered 100%, and all activities of the mutant TnT thin filaments were normalized to this value.

### Unloaded in vitro motility

The basic method and analysis followed our previously described motility assay using the anti-GFP attachment method [37] with the following modifications for thin filaments [46], [47]. Fluorescently-labeled thin filaments were prepared one hour before use by mixing constituents in the following concentrations: 2 µM actin labeled with tetramethylrhodamine (TMR)-phalloidin, 0.5 µM tropomyosin, and 0.5 µM troponin complex. The assay buffer (AB) used was 25 mM Imidazole (pH 7.5), 25 mM KCl, 4 mM MgCl_2_, 2 mM EGTA, 4 mM NTA, 1 mM DTT, and CaCl_2_ for a final pCa of 4. Anti-GFP antibody, myosin, and free Ca^2+^ concentrations were varied to determine the conditions that supported maximal velocity. The myosin concentration flowed through the chamber was 0.1 to 0.3 mg/ml. The final motility solution of AB with 1 mg/mL BSA (ABBSA) contained methylcellulose at a concentration of 0.5%, (TMR)-phalloidin labeled thin filaments, 50 nM excess troponin complex, 50 nM excess tropomyosin, 2 mM ATP, an oxygen scavenging system (0.3–0.4% glucose, 0.25 µg/mL glucose oxidase, 0.45 µg/mL catalase), and an ATP regeneration system (1 mM phosphocreatine, 0.1 mg/mL creatine phosphokinase). All thin filaments were checked in the absence or presence of saturating Ca^2+^ concentration to confirm that the filaments were fully regulated and either completely stopped or moving, respectively. Movies of sixty seconds were obtained at 23°C at a frame rate of 1 Hz using a Nikon Ti-E inverted microscope with Andor iXon + EMCCD camera model DU885.

Actin filament tracks were identified using Matlab FIESTA [48], followed by threshold scoring as previously described [37]. Stringent quality standards were maintained during motility experiments. To eliminate inactive or damaged myosin S1 heads that otherwise slowed down motility, we used a well-known method termed “dead-heading” [37]. For each S1 preparation this was performed multiple times until all fluorescent actin filaments were moving smoothly in the motility assay and there were no observed stuck or slowed filaments. Once there was smooth pure actin filament movement, we then collected data for thin filaments under the same conditions. Stringent requirements for motor quality were critical to obtain the fastest speeds as a small proportion of rigor motor easily slowed speeds >10%.

### ANS troponin complex

The triple TnC mutant containing one cysteine (C35S, T53C, C84S) was dialyzed against 50 mM Tris, pH 7.5, 100 mM KCl, 1 mM EGTA, as previously described [36]. Two to threefold excess anilinonapthalenesulfote iodoacetamide (IAANS) was added, and labeling proceeded in the dark at 4°C with gentle rocking for 2–5 hours. The reaction was stopped by the addition of 2 mM DTT, and unreacted IAANS was removed by dialysis. The concentration and percent labeling were determined using the Bradford assay and the extinction coefficient for ANS at 325 nm of 24,900 M^−1^cm^−1^, respectively. Iodoacetamide is known to also label lysine residues with lower reactivity, so single labeling was confirmed through mass spectrometry. The activity of the thin filament containing labeled TnC was similar to the unlabeled sample in both in vitro motility and pCa ATPase assays.

ANS-labeled TnC steady state assays were performed in a Fluorolog fluorimeter (Horiba Scientific) at 23°C, similar to previous studies [36], [49]. Samples were excited at 330 nm and emission was monitored at 450 nm. The excitation and emission bandwidths were kept at 7 nm for all experiments. Thin filaments were prepared at a ratio of 7∶1∶0.6–0.7 actin:tropomyosin:troponin where actin was 1.4 µM, tropomyosin 0.2 µM, and troponin complex 0.12–0.14 µM. To limit any free complex, troponin complex was added at sub-saturating concentrations. Final buffer conditions were 200 mM Hepes (pH 7.5), 10 mM KCl, 4 mM MgCl_2_, 1 mM DTT, 4 mM NTA, and 2 mM EGTA. Similar to the ATPase assays, the amount of CaCl_2_ needed for each Ca^2+^ concentration was determined using the pCa calculator [45]. The high buffer concentrations were used to limit any changes in pH upon CaCl_2_ addition. For cycling S1 experiments, ATP was added to 2 mM and an actin to S1 ratio of 1∶0.1 was used at an S1 concentration of ∼0.14 µM.

Transient Ca^2+^ dissociation rates were measured using a HiTech SF-61DX2 (TgK Scientific Ltd., U.K.) stopped-flow apparatus at 23°C. ANS fluorescence was excited at 330 nm and monitored using a 455-nm long pass (GG455) emission filter. The buffer used was 200 mM Hepes (pH 7.5), 10 mM KCl, 3 mM MgCl_2_, and 1 mM DTT. Troponin complexes or thin filaments were prepared at the same ratio as were used for the steady state experiments with 0.2 mM CaCl_2_ and then rapidly mixed with 20 mM EGTA to measure the effective Ca^2+^ dissociation rate. The initial concentrations were 10.5 µM actin, 1.5 µM tropomyosin, and 1.2 µM troponin complex. Each data trace was individually fit to a single exponential, with more than five individual traces for each mutant.

### Actin Pyrene Labeling

Actin was labeled similarly to previous methods [50]. Briefly, F-actin was dialyzed into 50 mM Hepes, pH 7.5, 100 mM KCl, and 0.2 mM CaCl_2_ at 4°C. The F-actin was labeled with fivefold excess pyrene-maleimide overnight at 23°C in the dark. The reaction was quenched with tenfold excess DTT, and undissolved pyrene was sedimented in a tabletop centrifuge at 11,000×g for 15 minutes. The F-actin was then cycled into G-actin at 4°C (G-buffer: 2 mM Tris, pH 8, 0.2 mM ATP, 0.2 mM CaCl_2_, 1 mM DTT). Any remaining F-actin was pelleted at 38,000×g for one hour at 4°C. Pyrene-labeled G-actin was dialyzed into 20 mM Hepes, pH 7.5, 10 mM KCl, 2 mM MgCl_2_, and 1 mM DTT and used within a week before recycling the actin again from F to G. The concentration and percent labeling of G-actin was determined by measuring the Bradford assay for protein concentration and the extinction coefficient of pyrene at 344 nm (22,000 M^−1^ cm^−1^) (5).

### ADP Release Rate

Maximal Ca^2+^-activated ADP release rates were measured by stopped-flow at 23°C in 25 mM Hepes (pH 7.5), 25 mM KCl, 4 mM MgCl_2_, 0.2 mM CaCl_2_, and 1 mM DTT. For each mutant, ≥6 traces were collected and fit individually. Regulated thin filaments were mixed with S1-ADP to a final concentration of 2 µM pyrene-actin, 0.5 µM tropomyosin, 0.5 µM troponin complex, 2 µM S1, and 50 µM ADP. After an incubation of at least 5 minutes, thin filaments were rapidly mixed with buffer containing 2 mM ATP and 50 µM ADP.

### Tropomyosin pyrene labeling

Tropomyosin pyrene labeling was performed under denaturing conditions similar to previous methods [51], [52]. Tropomyosin was dialyzed into 50 mM Tris, pH 6.5, 0.3 mM EDTA, and 5 M guanadinium hydrochloride at 23°C. Tropomyosin was labeled in the dark with 5 M excess pyrene-malemide overnight at 37°C. The reaction was quenched with 10 M excess DTT. After centrifuging the tropomyosin to clarify it of any undissolved pyrene, the pyrene labeled tropomyosin was dialyzed into the same buffer as above with added 1 mM DTT, at pH 8.4 and 23°C. By increasing the pH to 8.4, the succinimido ring opens up by aminolysis [53] and increases the pyrene signal [52]. The pH of the dialysis buffer was then adjusted to 7.5 at 23°C and the pyrene labeled tropomyosin dialyzed for at least 12 hours. The concentration and percent labeling of tropomyosin was determined by measuring the Bradford assay for tropomyosin concentration using a tropomyosin standard and the extinction coefficient of pyrene at 344 nm (22,000 M^−1^ cm^−1^). All binding experiments were carried out with 100 nM pyrene-tropomyosin at 23°C in 20 mM Hepes, pH 7.5, 100 mM KCl, 2 mM MgCl_2_, and 1 mM DTT.

## Results

For this study, we used thin filaments composed of the human cardiac troponin complex, bovine cardiac tropomyosin, and chicken skeletal actin (Fig. 1B). Human tropomyosin expressed in *E. coli* expressing N-α-acetyltransferase is not fully acetylated [54], which is required for appropriate tropomyosin–actin filament assembly. One can add a couple of residues to mimic the acetylation [55], but we chose instead to use bovine cardiac tropomyosin, which is acetylated, and differs from human tropomyosin by only two very conservative residue changes. Actin is one of the most conserved proteins known, and chicken skeletal actin, which is easy to obtain in large quantity, differs from human cardiac actin by only four conservative residue changes. We therefore have used chicken skeletal actin in these studies. We have recently carried out experiments with bovine cardiac actin (which is identical to human cardiac actin) and observed no differences as compared to these studies using chicken skeletal actin (data not shown). For all assays, we used a subfragment 1 (S1) construct of human β-cardiac myosin containing a truncated human cardiac myosin heavy chain (residues 1-808) and the human ventricular essential light chain (ELC) (Fig. 1A).

### The affinity of the troponin complex for tropomyosin is not affected by the TnT mutations

**Figure 2:**
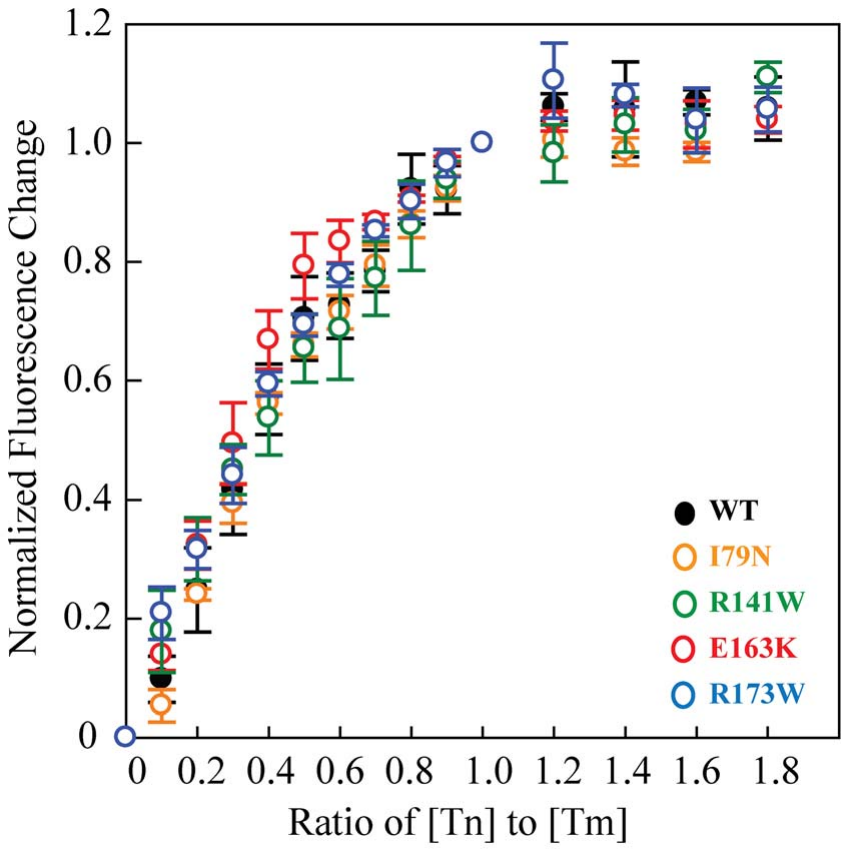
Binding of troponin complex to pyrene-labeled tropomyosin at 23°C. Each curve is the average of ≥ three individual curves, and error bars represent SEM. Buffer conditions were 20 mM Hepes, pH 7.5, 100 mM KCl, 2 mM MgCl_2_, 1 mM DTT. WT is in black, I79N in orange, R141W in green, E163K in red, and R173W in blue.

The four mutations examined in this study are located within or adjacent to the tropomyosin binding region of TnT (Fig. 1C and 1D). We first examined whether the affinity of the troponin complex for tropomyosin was affected. Studies with pyrene-labeled tropomyosin showed no significant changes in tropomyosin binding affinity for the mutant troponin complexes compared to WT. The binding affinities (K_d_) range from 70−120±10−40 nM (Fig. 2) and are similar to previous measurements for skeletal troponin complex binding to tropomyosin [56].

### TnT mutations have no effect on the rate of maximal Ca^2+^-activated ADP release from the human β-cardiac S1-thin filament complex

Earlier studies of HCM- and DCM-causing troponin-T mutations have shown changes in the maximal sliding velocities of skeletal muscle HMM under low load, suggesting changes in the myosin ADP release kinetics (e.g. [15], [24], [57], [58]). The major difference between those studies with skeletal muscle HMM and those reported here is that the motor domains of skeletal myosin and human β-cardiac myosin differ by >150 residues. Using human β-cardiac S1, we examined the effect of four TnT mutations on maximal Ca^2+^-activated ADP release kinetics using both in vitro motility and transient kinetics experiments. In the in vitro motility assay, the average maximal sliding velocity (*υ_0_*) is related to the myosin stroke size *d* and *t_s_* or duration of time that the myosin remains strongly attached to the thin filament, i.e. *υ_0_ = d/t_s_* [59]. Any changes in maximal velocity likely reflect changes in *t_s_*, which for cardiac myosin is determined by the ADP release rate [60]. In contrast to previous studies [15], [24], [61], we observed no significant change between the WT maximal sliding velocity and each of the mutants at saturating Ca^2+^ using human β-cardiac S1 (Table 1), suggesting no change in maximal ADP release rate.

**Table 1:** Summary of the maximal Ca^2+^ activated in vitro motility velocities and ADP release rates at 23°C with human β-cardiac S1.

TnT	Disease	Velocity at 23°C (nm s^−1^) (*n*)	ADP release rate at 23°C (*n*) (s^−1^)
WT		550±20 (*7*)	67±1 (*8*)
I79N	HCM	490±20 (*5*)	66±1 (*6*)
E163K	HCM	600±40 (*6*)	68±1 (*7*)
R141W	DCM	520±20 (*5*)	69±1 (*7*)
R173W	DCM	520±10 (*3*)	67±1 (*8*)

Mean ± SEM.

*n*, represents number of motor preparations for in vitro motility velocities, and number of kinetic traces for ADP release rate.

Number of filament tracks: WT, 1401; I79N, 1190; E163K, 1307; R141W, 1037; R173W, 827.

All p-values for mutant vs WT thin filament velocities were ≥ 0.05 and hence not significant.

To confirm the lack of change in ADP release rate directly, we measured the ADP release rates using stopped flow kinetics at saturating Ca^2+^ concentration. For both WT and mutant troponin T thin filaments, we measured ADP release rates of ∼67 s^−1^ (Table 1). This is similar to the ADP release measured with unregulated actin under the same conditions (∼72 s^−1^). These results, along with the motility velocities, suggest that the maximal Ca^2+^-activated ADP release kinetics of human β-cardiac S1 are unaffected by these point mutations in TnT, contrary to results from previous studies with non-human skeletal myosins.

### HCM-causing mutations increase and DCM-causing mutations decrease Ca^2+^ sensitivity of actin-activated human β-cardiac S1 ATPase activity

**Figure 3:**
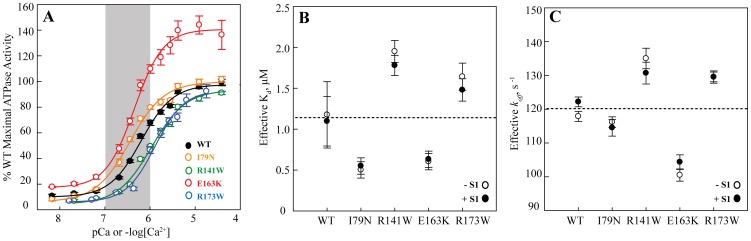
Ca^2+^ sensitivity of WT and mutant TnT thin filaments. (A) Ca^2+^ sensitivity of thin-filament-activated ATPase activity of human β-cardiac S1 at 30°C. Each curve is the average of >10 individual curves, and error bars represent SEM. The curves were individually fit to the Hill equation, and normalized to the maximum activity of WT, as summarized in Table 2. WT is in black, I79N in orange, R141W in green, E163K in red, and R173W in blue. The shaded gray region represents the approximate physiological Ca^2+^ range during a heartbeat (See Discussion). (B) The effective K_d_ for Ca^2+^ induced conformation changes with (filled circles) and without (empty circles) cycling S1 using ANS-TnC^T53C^ thin filaments at 23°C. Error bars represent SEM. The dotted line represents the average effective K_d_ for WT thin filaments. There was no significant difference with and without cycling motor (p>0.05). (C) The effective Ca^2+^ dissociation rates or *k_off_* with (filled circles) and without (empty circles) cycling S1 using ANS-labeled thin filament at 23°C. Error bars represent SEM. The dotted line represents the average effective *k_off_* for WT thin filaments. As with the K_d_'s, there was no significant difference in the effective *k_off_* with and without cycling motor (p>0.05).

To measure the effect of the TnT mutations on the Ca^2+^ sensitivity of the thin filament, thin filament-activated human β-cardiac S1 ATPase activity was measured as a function of free Ca^2+^ concentration. HCM-causing mutations I79N and E163K were sensitizing; they increased the pCa50 (−log [Ca^2+^] at half-maximal activation) by +0.27 and +0.19, respectively. The DCM-causing mutations R141W and R173W were desensitizing; they decreased the pCa50 by −0.21 and −0.09, respectively (Fig. 3A, Table 2). This trend is consistent with what has been previously observed for other HCM- and DCM-causing troponin mutations using non-human myosin [17].

**Table 2:** Summary of the thin filament activated ATPase pCa curves for human β-cardiac S1 at 30°C.

TnT	Disease	pCa50	ΔpCa50	% Maximum Activity	% Minimum Activity	n_H_	*n*
WT		6.17±0.01	−	100±1%	10±1%	1.2±0.1	33
I79N	HCM	6.44±0.04*	+0.27	100±2%	7±1%	1.2±0.1	11
E163K	HCM	6.36±0.03*	+0.19	147±7% *	15±1%	1.4±0.2	11
R141W	DCM	5.96±0.02*	−0.21	93±2% *	5±1%	1.2±0.1	11
R173W	DCM	6.08±0.01*	−0.09	101±4%	6±1%	1.5±0.1	15

Mean ± SEM.

n_H_ is the Hill coefficient.

*n*, represents number of individual ATPase pCa curves.

* , p<0.001 by Mann-Whitney Rank Sum Test.

From the ATPase pCa curves, we were also able to measure the effects of the TnT mutations on the maximal activation of the thin filament. While both I79N and R173W were similar to WT, the HCM-causing E163K mutation showed a statistically significant increase in maximal activity (∼47%, p<0.001), while the DCM-causing R141W mutation showed a statistically significant decrease (∼7%, p<0.001) (Table 2). These differences could potentially reflect a slight change in the affinity of the myosin towards the thin filament at saturating Ca^2+^.

### TnT mutations affect the inherent Ca^2+^ sensitivity of the thin filaments but not of the troponin complexes

To examine the inherent Ca^2+^ binding properties of the troponin complex and the thin filament, we studied the effects of the TnT mutations in the absence of myosin. We used a fluorescent ANS probe on a T53C modified TnC (TnC^T53C^, see Experimental Procedures) to monitor the changes in Ca^2+^ sensitivity. Previous work [36] to monitor the changes in Ca^2+^ sensitivity has shown that Ca^2+^ binding to the complex alone or in reconstituted thin filaments, causes either a decrease or increase in the ANS fluorescence, respectively. This indicates that changes in ANS environment are coupled to an overall conformational change in the troponin complex upon Ca^2+^ binding. For the thin filaments, this is also coupled to a shift in tropomyosin along actin. This signal therefore can be used to measure an ‘effective’ K_d_ for Ca^2+^-induced conformational changes. This conformational change is discussed further in later sections and data is shown in Table 3.

**Table 3:** Summary of Ca^2+^ sensitivity and properties of WT troponin complex at 23°C at various conditions.

Condition	pCa50	Eff K_d_ (μM)	n_H_	*n*
Tn Complex	8.19±0.23	0.007±0.003	−0.44±0.02	11
Tn Complex + Tm	8.05±0.1	0.009±0.002	−0.47±0.03	11
Thin filaments no S1	5.93±0.15	1.2±0.4	0.93±0.08	7
Thin filaments + cycling 0.1∶1 S1	5.96±0.12	1.1±0.3	1.53±0.39	8
Thin filaments + cycling 1∶1 S1	5.82±0.1	1.5±0.3	1.26±0.20	5
Thin filaments + 0.01∶1 rigor S1	6.29±0.03	0.51±0.04	1.33±0.10	3
Thin filaments + 0.1∶1 rigor S1	6.41±0.10	0.39±0.09	1.51±0.31	3
Thin filaments + 1–1.5∶1 rigor S1	6.85±0.02	0.14±0.01	−0.73±0.02	10

Mean ± SEM.

n_H_ is the Hill coefficient.

*n*, represents number of individual curves.

Ratios represent the molar ratio of S1 to thin filament.

**Figure 4:**
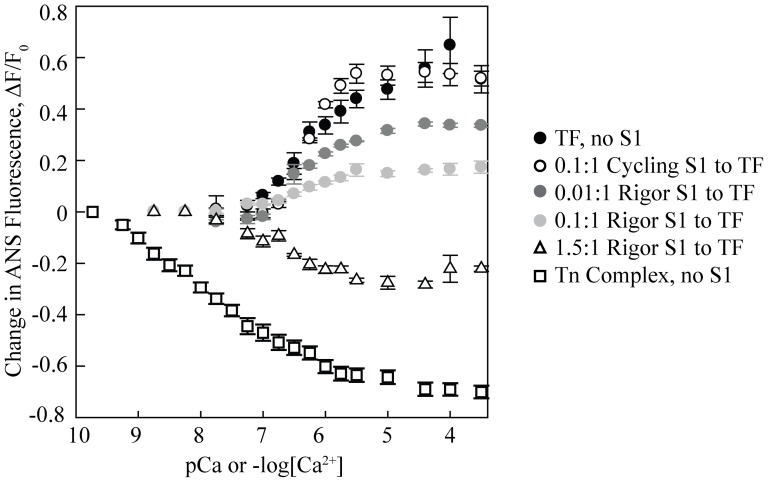
WT ANS-labeled troponin complex properties. Ca^2+^ sensitivity of WT-ANS troponin complex and thin filament with different amounts of human β-cardiac S1 at 23°C. Each curve is the average of at least three individual curves, and error bars represent SEM. Black filled circles, thin filament with no S1; open circles, 0.1∶1 cycling S1 to thin filament; dark grey filled circles, 0.01∶1 rigor S1 to thin filament; light grey filled circles, 0.1∶1 rigor S1 to thin filament; open triangles, 1.5∶1 rigor S1 to thin filament; open squares, troponin (Tn) complex with no S1. TF in the legend stands for thin filament.

We first examined the troponin complex alone. For both the WT and mutant troponin complexes, increasing Ca^2+^ concentration is associated with a decrease in fluorescence with a mid-point of fluorescence loss in the low nanomolar regime (Fig. 4). Similar results were observed in the presence of tropomyosin. Upon addition of actin, however, we observed changes in the effective K_d_ for the mutants (Fig. 3B). The pCa50 for WT was 5.9±0.2, which yields an effective K_d_ of 1.2±0.4 µM. For the two HCM mutants, the effective K_d_ was ∼2–2.4 fold lower than the WT while for the two DCM mutants the effective K_d_ was ∼1.4–1.7 fold higher than the WT. Together the steady state data imply that while the TnT mutations have no effect on the effective K_d_ for Ca^2+^-induced conformational changes of the troponin complex alone, in the context of the regulated thin filaments, the DCM mutants have a higher effective K_d_ as compared to the WT and the HCM mutants have a lower effective K_d_. This indicates that the inherent Ca^2+^ sensitivity and/or the associated conformational changes in the regulatory proteins are affected by mutations in TnT, such that HCM mutants show a sensitizing effect and vice-versa for the DCM mutants.

### TnT mutations alter the kinetics of dissociation

The effective conformational *k_off_* from the thin filament for the WT and four disease-causing TnT mutants were examined using transient kinetic measurements of the ANS-TnC^T53C^ probe (Fig. 3C). For the WT thin filament the effective *k_off_* was 118±1 s^−1^, which was similar to that for the HCM mutant I79N (116±2 s^−1^). However for E163K (101±2 s^−1^) the effective *k_off_* was ∼15% lower than the WT. For both R141W (135±3 s^−1^) and R173W (130±2 s^−1^) the effective *k_off_* was ∼10–15% greater than the WT. The general trend of higher *k_off_* values for the DCM mutants is consistent with them being desensitizing and vice versa for the HCM mutants.

### Cycling human β-cardiac S1 has no effect on the Ca^2+^ sensitivity of the regulated thin filament

It is widely accepted that cardiac myosin cross-bridges can have substantial effects on the Ca^2+^ sensitivity of the cardiac thin filaments ([62], see Discussion). We therefore studied the TnT mutant thin filaments by steady state fluorescence measurements in the presence of human β-cardiac S1 and physiologically relevant ATP concentrations (2 mM). There was no significant change in the effective K_d_ values compared to those in the absence of myosin with either 0.1∶1 or 1∶1 cycling S1 to thin filament (Fig. 3B), though the Hill coefficient (n_H_) did marginally increase from ∼1 to ∼1.4. We additionally measured the effect of cycling motor on the kinetics of conformational changes in the thin filaments upon Ca^2+^ dissociation (Fig. 3C). Consistent with the steady state data, there was no significant difference with and without cycling motor, and the trend for the HCM- and DCM-causing mutations was maintained. This data is not consistent with previous reports (done using skeletal muscle components and non-physiological rigor cross-bridges [62]) that suggest that the presence of cycling cross-bridges increases the Ca^2+^ sensitivity of the thin filaments [63].

In order to further understand the role of cycling and rigor cross-bridges we further investigated the Ca^2+^ induced ANS fluorescence changes in the presence of cycling and rigor motors. As shown in Fig. 4 and Table 3, in the presence or absence of cycling myosin, the WT thin filament fluorescence changes were similar in magnitude and direction showing increased fluorescence with increasing Ca^2+^ concentration. A similar trend was observed at sub-saturating rigor myosin concentrations, although the magnitude of the change decreased as the amount of rigor motor increased. The pCa50 also increased with increasing ratios of rigor S1 to thin filaments. At saturating rigor myosin concentrations, the ANS fluorescence dramatically decreased with increasing Ca^2+^. At these concentrations of rigor S1, the TnT mutant thin filaments no longer showed any differences from WT.

## Discussion

The majority of in vitro biochemical and biophysical experiments on thin filaments have been carried out with bovine or porcine β-cardiac myosin constructs (ATPase), which differ by >30 residues from human β-cardiac myosin, or with rabbit skeletal HMM (motility), which differs by >150 residues. When comparing skeletal and β-cardiac muscle myosins, they also have distinct biochemical and kinetic properties [27]. For example, in skeletal muscle, myosin ADP release is extremely fast as is the rate of cross-bridge detachment, which is likely limited by the rate of ATP binding. With β-cardiac myosin, on the other hand, ADP release is much slower and is the rate-limiting step for cross-bridge detachment [27]. Considering these differences, this study reexamines the effects of cardiomyopathy-causing thin filament mutations with human β-cardiac myosin S1.

Ca^2+^ and the position of tropomyosin regulate the rate of strong binding of myosin to the thin filament by a steric blocking mechanism. This has been supported by a number of biochemical studies, including work showing the Ca^2+^ sensitivity of in vitro motility velocities [62], 64. One question, though, has been whether cardiomyopathy-causing troponin mutations can alter the maximal Ca^2+^-activated myosin ADP release cross-bridge kinetics. Previous studies using skeletal muscle HMM showed changes in the maximally activated sliding velocity for the I79N and R141W mutations [15], [24], [61], suggesting differences in the maximally activated ADP release rates. Using human β-cardiac S1, we did not observe these changes for any of the mutant troponin thin filaments as compared to WT. Our results suggest that the inherent actomyosin ADP release kinetics are not altered by these mutations in TnT at saturating Ca^2+^ concentrations.

As has been seen in previous studies with non-human myosins, our data with human β-cardiac S1 show that the HCM-causing TnT mutations result in increased Ca^2+^ sensitivity and the DCM-causing mutations in decreased sensitivity. According to the classical ‘three state model’ of muscle regulation, tropomyosin blocks myosin binding sites on the actin in the absence of Ca^2+^ (the ‘blocked’ state). Myofilament activation subsequently occurs via Ca^2+^-induced changes in the troponin complex, causing a significant azimuthal shift in the position of tropomyosin (the ‘closed’ state). Myosin cross-bridges then further push tropomyosin into an ‘open’ state to form a fully activated myofilament [65]. Hence, both the intrinsic Ca^2+^ binding properties of TnC followed by associated conformation changes in the thin filament proteins play an important role in muscle regulation. The effective K_d_ measured in this study, therefore, is likely a convolution of the rates of these steps.

Even though the four mutations examined in this study are located within or adjacent to the tropomyosin binding region of TnT, we observed no changes in tropomyosin binding affinities for the mutant troponin complexes compared to WT. The changes we see in the effective Ca^2+^-induced conformational change kinetics therefore likely reflect the changes in the rates of either Ca^2+^ binding and/or tropomyosin conformational dynamics. In the case of our HCM-causing mutations, the data suggest a decrease in the rate of closing of the thin filament, which would result in an increased Ca^2+^ sensitivity. The opposite effects held true for both DCM-causing mutations. Similar results were observed in the presence of cycling human β-cardiac S1.

As the effect of cycling cross-bridges on Ca^2+^ affinity in cardiac muscle has been somewhat debated in the literature [62], it was important to test using human β-cardiac myosin. One study in bovine cardiac muscle fibers, for example, showed that rigor cross-bridges significantly increased Ca^2+^ sensitivity, and cycling cross-bridges had similar effects [63]. A more recent study using cardiac thin filaments and skeletal S1 showed an increase in the Ca^2+^ sensitivity (decrease in K_d_) with rigor cross-bridges though not with cycling cross-bridges. They did see, though, some changes in the dissociation rates (*k_off_*) with cycling S1 for both WT and HCM-causing troponin mutant thin filaments [66]. Using skeletal S1 and WT cardiac thin filaments, Davis et al. showed an increased Ca^2+^ sensitivity (decrease in K_d_) with rigor S1, but they saw no change in either K_d_ or *k_off_* with cycling cross-bridges [36]. With human β-cardiac S1, our results with both rigor and cycling S1 agree with the results from Davis et al. [36], for both WT and HCM- and DCM-causing mutations. The behavior of the six component regulatory system is very different in the presence of cycling and rigor myosin. One possible explanation is that rigor myosin pushes the tropomyosin into the ‘open’ state such that at high enough concentrations, the conformational change observed upon Ca^2+^ binding is mostly due to internal rearrangement in the troponin complex.

Overall, when the heart beats, it continuously cycles through systolic contraction and diastolic relaxation. Ca^2+^ is key to this, as the Ca^2+^ binding to TnC opens up the thin filament, allowing myosin to interact and create force-producing cross-bridges [67]. In the sarcomere, this relationship between Ca^2+^ activation and myosin activity is finely tuned. During the heartbeat, the myoplasmic Ca^2+^ concentration fluctuates between ∼10^−7^ M to only ∼10^−6^ M [7], [8] (shaded region in Fig. 3A). This means that the thin filament is never actually maximally activated during contraction. Any changes in the Ca^2+^ sensitivity would therefore have significant effects on the number of force producing myosin cross-bridges and the overall sarcomeric force. The increased sensitivity from HCM-causing mutations should increase the number of cross-bridges, predicting a higher force producing state of the heart. This could also lead to diastolic problems, as the heart may not be able to fully relax. Both of these are features of the pathophysiology of HCM [68]–[70]. The decreased sensitivity of the DCM-causing mutations, on the other hand, would lead to fewer myosin cross-bridges at normal physiological Ca^2+^ levels and a lower force producing state of the cardiac muscle, and indeed impaired systolic function is the clinical hallmark of DCM [4], [71].

While our results with human β-cardiac S1 agree with previous studies using non-human myosin constructs in terms of thin filament sensitivity, we saw differences from previous studies using skeletal myosin HMM when examining myosin-thin filament cross-bridge kinetics. Future work will expand on these studies using more complex reconstitutions, including full length myosin or HMM constructs and regulation of the regulatory light chain by phosphorylation. A future challenge will be to connect these underlying kinetic changes with the pathophysiological changes observed in patients with HCM and DCM.
